# Detection of Tick-Borne Pathogens of the Genera *Rickettsia, Anaplasma* and *Francisella* in *Ixodes ricinus* Ticks in Pomerania (Poland)

**DOI:** 10.3390/pathogens10070901

**Published:** 2021-07-15

**Authors:** Lucyna Kirczuk, Mariusz Piotrowski, Anna Rymaszewska

**Affiliations:** 1 Department of Hydrobiology, Faculty of Biology, Institute of Biology, University of Szczecin, Felczaka 3c Street, 71-412 Szczecin, Poland; lucyna.kirczuk@usz.edu.pl; 2 Department of Genetics and Genomics, Faculty of Biology, Institute of Biology, University of Szczecin, Felczaka 3c Street, 71-412 Szczecin, Poland; mariusz.piotrowski@bio-space.pl

**Keywords:** *Rickettsia helvetica*, tick-borne pathogens, *Anaplasma phagocytophilum*

## Abstract

Tick-borne pathogens are an important medical and veterinary issue worldwide. Environmental monitoring in relation to not only climate change but also globalization is currently essential. The present study aimed to detect tick-borne pathogens of the genera *Anaplasma*, *Rickettsia* and *Francisella* in *Ixodes ricinus* ticks collected from the natural environment, i.e., recreational areas and pastures used for livestock grazing. A total of 1619 specimens of *I. ricinus* were collected, including ticks of all life stages (adults, nymphs and larvae). The study was performed using the PCR technique. Diagnostic gene fragments *msp2* for *Anaplasma*, *gltA* for *Rickettsia* and *tul4* for *Francisella* were amplified. No *Francisella* spp. DNA was detected in *I. ricinus.* DNA of *A. phagocytophilum* was detected in 0.54% of ticks and *Rickettsia* spp. in 3.69%. Nucleotide sequence analysis revealed that only one species of *Rickettsia*, *R. helvetica*, was present in the studied tick population. The present results are a part of a large-scale analysis aimed at monitoring the level of tick infestation in Northwest Poland.

## 1. Introduction

Blood-sucking arthropods, such as ticks, insects and mites, are a group that plays a special role in the spread of many species of obligate intracellular microorganisms. Among them, hard ticks of the family Ixodidae, are very important as cosmopolitan vectors of pathogens that cause disease in humans and domestic animals. *Ixodes ricinus* is one of the most important species of hard ticks that plays a significant role in the transmission of bacteria, viruses and protozoa. The pathogens transmitted by ticks include bacteria of the genera *Borrelia, Anaplasma, Ehrlichia, Rickettsia* and *Francisella* and protozoa of the genera *Babesia* and *Theileria* [1,2]. 

Bacteria of the genera *Anaplasma* and *Rickettsia* belong to obligate intracellular pathogens [1,2], while *Francisella* is a facultative intracellular pathogen [3]. All are pathogens of vertebrates and have a wide range of species as their reservoirs. These host species include, among others, deer, wild boar, small rodents and birds [4,5,6,7]. 

Microorganisms belonging to the family *Anaplasmataceae* are intracellular *alpha-1* proteobacteria that multiply in the vacuoles of eukaryotic cells. According to the latest classification, this family includes five genera—*Anaplasma*, *Ehrlichia*, *Aegyptianella*, *Neorickettsia* and *Wolbachia*—of which *Anaplasma* and *Ehrlichia* are considered as the most pathogenic [1,8]. *Anaplasma phagocytophilum* causes human granulocytic anaplasmosis (HGA) in humans and tick-borne fever (TBF) in many domestic animals, mainly small ruminants, horses and dogs [2,6].

Bacteria belonging to the genus *Rickettsia* comprise two historical groups: the typhus group (TG) and the spotted fever group (SFG), and two recently separated new groups: the transitional group (TRG; *R. felis*, *R. akari* and *R. australis*), and the ancestral group (AG; *R. bellii* and *R. canadensis*) [9,10]. For *Rickettsia*, ticks are not only efficient vectors but also competent reservoirs. Rickettsioses, like anaplasmosis, pose an important medical and veterinary problem. In the description of the disease, the classical symptomatic triad of fever, rash and headache, which are the main clinical signs to diagnose rickettsiosis but are also observed in other tick-borne diseases, is given as a delineation. Each instance of rickettsiosis has been shown to have specific characteristics, including severity. Rickettsioses can be mild, severe or even fatal [7,10].

Tularemia is an acute zoonotic disease caused by the Gram-negative aerobic bacillus *Francisella tularensis*. There are four subspecies of *F. tularensis*, two of which are considered pathogenic for humans and animals: type A, *F. tularensis* subsp. *Tularensis*, and type B, *F. tularensis* subsp. *holarctica*. Type A is highly infectious and virulent, and therefore, remains an epidemiological issue in some countries. Type B is milder and occurs mainly in Europe and Asia. In Europe, type B has most been reported in Scandinavian countries, where it has remained stable since 2006 [5,11,12]. In Poland, there has been a small but stable increase in the incidence of tularemia. The first case of tularemia was diagnosed in 1949, and since then, more than 600 cases have been reported, mainly in the northeastern and northwestern regions of the country. In most cases, the infection was transmitted by ixodid ticks or by direct contact with infected animals [13]. Furthermore, members of the *Francisella* genus are highly pathogenic with a potential risk of bioterrorism. *Francisella tularensis*, along with *Bacillus anthracis, Yersinia pestis* and *Brucella* sp., is among the microorganisms that can be used for bioterrorist attacks; therefore, strict environmental monitoring is required to take appropriate and timely action [14].

The incidence of tick-borne diseases in Europe is lower than that reported globally. A feature of many of these infections in the Old Continent is their relatively mild course. The main symptoms of these infections are fever, headache and muscle and joint pains, which resemble the common cold. However, monitoring of ticks reveals the presence of pathogens, which is not reflected in the dynamics of human infections. Hence, it appears that tick-borne diseases may be underdiagnosed or underreported in European countries [15]. In Poland, as in many other countries, it is mandatory to report and register tick-borne diseases [15,16].

Despite many years of research, the topic of screening of questing ticks that are major vectors of tick-borne pathogens is still relevant. Molecular research of ticks collected from vegetation is part of environmental monitoring. Information on ticks infected by pathogens is important for medical and veterinary doctors. Monitoring of ticks for the presence of pathogens facilitates the diagnosis of humans and animals. The present study aimed to estimate the infection of the castor bean tick *I. ricinus* captured from the environment by *A. phagocytophilum, Rickettsia* spp. and *Francisella* spp. The areas designated for sample collection were recreational sites and meadows used for livestock grazing. 

## 2. Results

**Figure 1:**
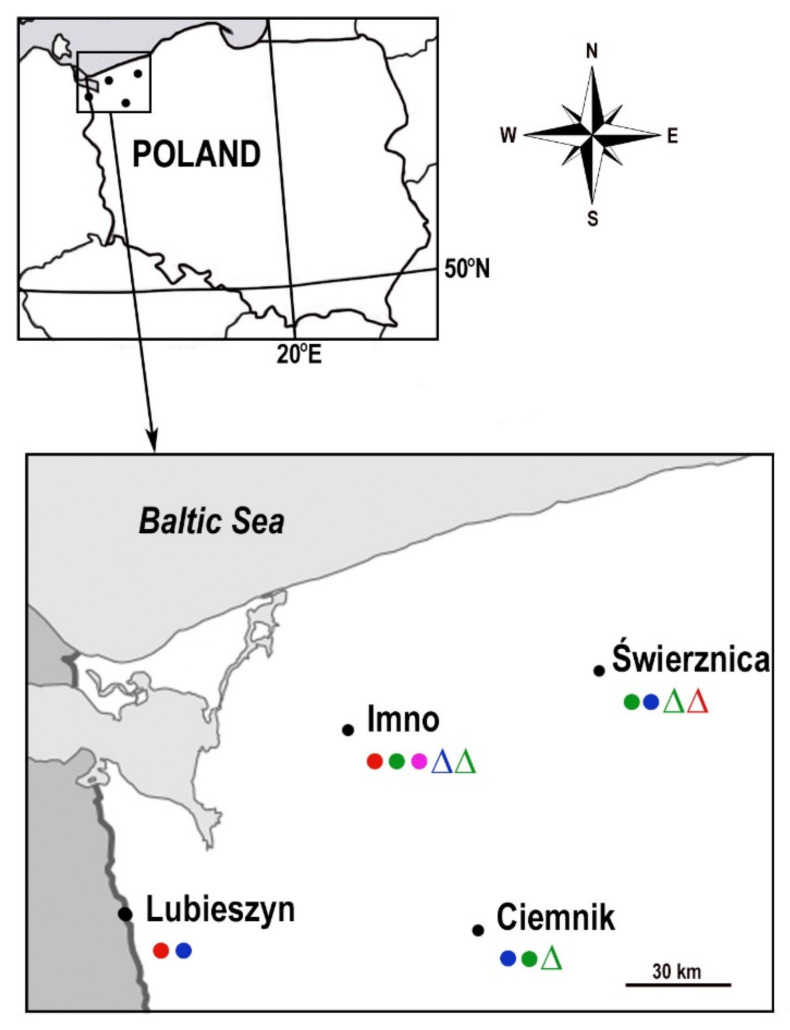
Collection sites of *Ixodes ricinus.* Tick infection: ● *R. helvetica* (male ticks), ● *R. helvetica* (female), ● *R. helvetica* (nymph), ● *R. helvetica* (larvae); ∆ *A. phagocytophilum* (male), ∆ *A. phagocytophilum* (female), ∆ *A. phagocytophilum* (nymph).

A total of 1682 *I. ricinus* ticks (111 females, 68 males, 1008 nymphs and 495 larvae) were collected from all sites (Figure 1, Table 1). None of the sites explored showed the presence of *Francisella* spp. DNA in *I. ricinus*. Overall, higher infection of ticks by *Rickettsia* than by *A. phagocytophilum* was observed. For *Rickettsia*, infection was found in two individuals (0.76%) in Lubieszyn and 24 (5.74%) in Imno. *Anaplsma phagocytophilum* was not found in Lubieszyn, while in Imno and Świerznica, anaplasma DNA was detected in four individuals, of 0.84 and 0.95%, respectively. In Ciemnik, only one nymph (0.19%) of 524 *I. ricinus* individuals was positive for *A. phagocytophilum*. Detailed infection data are provided in Table 2. Only one individual, a nymph of *I. ricinus* (0.06%), collected at the Świerznica site, was found to be co-infected with *A. phagocytophilum* and *R. helvetica.*

The presence of two nucleotide variants was observed in *A. phagocytophilum*, which has previously been described in ticks and has been reported to GenBank by the author [17]. The sequences of the *msp2* amplified fragment are identical to those of accession numbers DQ105670 (host *Capreolus capreolus*) and DQ105673 (host *I. ricinus*). Due to the identity of the nucleotide sequences generated in this study, they were not submitted to GenBank. Polish *A. phagocytophilum* sequences for the *msp2* gene fragment in comparison with sequences from farm animals, ticks (from Europe) and a human (US patient) showed an identity of more than 99% (data not shown).

**Figure 2:**
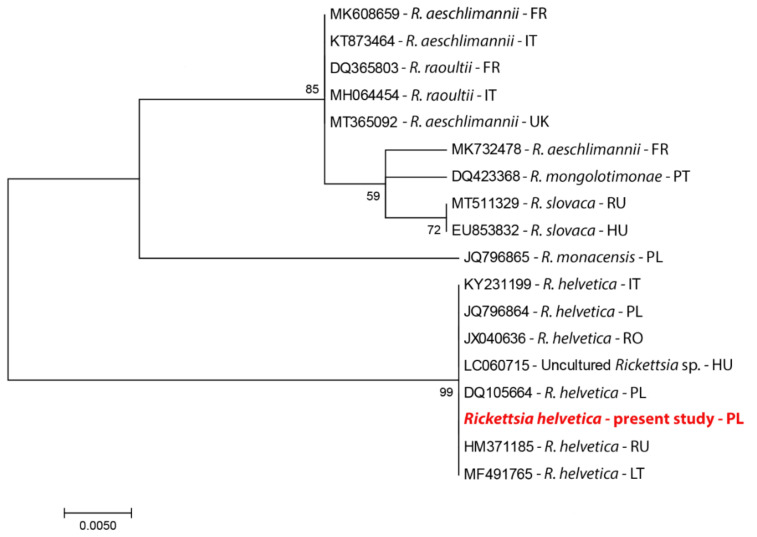
A phylogram constructed on the basis of the *gltA* gene. The analysis was performed on species of the genus *Rickettsia*, available from GenBank (country codes: https://www.pit.pl/intrastat/kody-panstw-intrastat-922638 accessed on 15 May 2021).

For *Rickettsia*, only one species, *R. helvetica*, was found based on the nucleotide sequence of the *gltA* gene, which is a good tool for taxonomic identification of bacteria [18]. Analysis of the *htrA* gene confirmed species affiliation for the studied isolates. No genetic variation was observed among the analyzed *gltA* and *htrA* genes. On the dendrogram, the obtained sequences for *R. helvetica* clustered with other sequences of this species obtained from ticks or reservoir species (rodents and dogs) in European countries (Figure 2).

## 3. Discussion

In Poland, the most commonly reported tick-borne diseases include Lyme disease and tick-borne encephalitis. Data from the National Institute of Hygiene (NIH) in 2019 show that 20,630 cases of Lyme borreliosis were registered, of which 1701 people were hospitalized. Moreover, in 2019, 265 cases of tick-borne encephalitis were registered, 68 more than in 2018. In contrast, 21 cases of tularemia (16 hospitalized) or four cases of rickettsiosis were reported in 2019, i.e., five less than in the previous year (http://wwwold.pzh.gov.pl accessed on 15 May 2021).

In Poland, epidemic outbreaks of tularemia occur mainly in the north and east of the country, i.e., in the region of Szczecin, Gdańsk, Bydgoszcz and Białystok. According to the Polish National Institute of Hygiene (NIH), the highest number of cases [8] was reported in Western Pomerania, and isolated cases were common in other regions. The area of Pomerania covered by the screening study did not show any *Francisella* sp. DNA in ticks. Wójcik-Falata et al. [19] studied *I. ricinus* and *Dermacentor reticulatus* ticks and revealed the presence of *F. tularensis* subspecies *holarctica* only in *Dermacentor* and at a very low level, only 0.2%. The role of *I. ricinus* in vectoring *Francisella* sp. is controversial. Transmission of these bacteria by *I. ricinus* has not been confirmed in Hungary, Luxembourg or France [20,21,22,23]. However in central Germany ranged from 1.2–1.5% for ticks feeding on birds and rodents to 1.6% for host-seeking ticks [4]. Higher levels of *I. ricinus* infection with *F. tularensis* were reported in Serbia (3.8%) [24] and in south-western Germany (8.4%); Some authors also indicated a higher level of infection for ticks of the genus *Dermacentor* [25,26]. On the basis of the analysis of the epidemiological status of tularemia in European countries, it seems that ticks do not play an important role in the transmission of *F. tularensis*, and infections of humans or domestic animals result mainly from contact with infected rabbits, hares, rodents or squirrels [5].

Regarding both granulocytic anaplasmosis and rickettsioses caused by SFG rickettsieae, the occurrence of the diseases in a given area is primarily associated with contact with infected ticks. In Europe, infection of ticks by *A. phagocytophilum* is low, ranging from 0.6% to 3% [20,21,27]. 

In the present study, the infection of ticks by both pathogens, *A. phagocytophilum* and *R. helvetica*, in northwestern Poland is comparable to the results reported in other European countries. In this part of Europe, ticks are mainly infected with *A. phagocytophium* and at low levels. Several species of *Rickettsia* spp. may occur, but the most common one is *R. helvetica*. In our study, we expected to detect two species of *Rickettsia* from the SFG, especially because the presence of *R. monacensis* in questing ticks of *I. ricinus* collected from the forests of Western Pomerania was reported [28]. However, nucleotide sequence analysis revealed that only *R. helvetica* was present in the examined tick populations from four sites in Western Pomerania. The latest research by Kowalec et al. [29] and Michalski et al. [30] conducted in *I. ricinus* ticks collected from central and northeastern Poland showed the presence of the species *R. raoulti* and the novel “*Candidatus Rickettsia mendelii*” in these ticks, apart from *R. helvetica* and *R. monacensis*. The authors’ current research, unpublished (material harvested in 2019–2020), in ticks collected from urbanized areas of Western Pomerania has not yet revealed the presence of species other than *R. helvetica* and *R. monacensis.*

Comparison of sequences obtained from *A. phagocytophilum* revealed high identity with sequences previously obtained from isolates from host-seeking ticks and ticks parasitized on wildlife [6,17]. Previous studies suggested that the nucleotide sequences of the two *A. phagocytophilum* variants detected did not cluster with the *msp2* gene sequences isolated from patients with suspected anaplasmosis.

As observed for tularemia, epidemiological data for the other two tick-borne diseases (spotted fever and anaplasmosis) do not correlate with the infection status of ticks. A very low incidence among humans has been observed, and at the same time, studies indicate infection of ticks, as well as the presence of antibodies in humans and animals considered potential reservoir species for these pathogens [31,32,33].

In Poland, over the past few years, rickettsiosis has been extremely rare, with less than 10 cases per year reported by the National Institute of Hygiene (http://wwwold.pzh.gov.pl accessed on 15 May 2021). However, tests for the presence of antibodies to tick-borne pathogens indicate frequent contact with bacteria of the genera *Rickettsia* and *Anaplasma.* The cases of tick-borne diseases with non-specific symptoms are underestimated. Information about a tick infection can be helpful for doctors and veterinarians. Therefore, regular monitoring of ticks should be carried out and it should cover urban areas.

In summary, it should be emphasized that *I. ricinus* ticks are a vector for *R. helvetica* and *A. phagocytophilum* in northwestern Poland. The search should be extended to include other transmitting or reservoir species for this pathogen.

## 4. Materials and Methods

### 4.1. Characteristics of the Collection Sites

Questing ticks were collected in July–August 2012 using the flagging method from grasslands in Western and Central Pomerania (Poland) (Figure 1), located near the villages of Świerznica (53.8597° N, 15.9962° E), Ciemnik (53.3833° N, 15.5667° E), Imno (53.5500° N, 14.9333° E) and Lubieszyn (53.4494° N, 14.3892° E). The areas selected for collection are characterized by high natural values. These places abound in green areas, including forests rich in vegetation, mainly deciduous and mixed forests. The village of Świerznica is located in the wooded valley of the Świerznica river, an area included in the special habitat protection area “Dorzecze Parsęty”. According to the Central Register of Nature Protection Forms (CRFOP) (data from 30 June 2017), there are six forms of nature protection in the area of Ciemnik village: Krzemieńskie Źródliska nature reserve, four nature monuments and Ostoja Ińska (special bird protection area—Natura 2000). The first and currently the largest studied farm of Shetland ponies in Poland is located in Imno. In the commune of Dobra, in which Lubieszyn is located (a hamlet of the village of Wąwelnica with a Polish-German border crossing), there are abundant forests covering the Wkrzańska Forest and the surroundings of the Świdwie Bird Nature Reserve (a breeding site of the white eagle). All these places serve as recreational sites for inhabitants and visitors, and they are particularly attractive as tourist sites for amateur walkers and cyclists who visit. Apart from value for tourism, a large part of the land in these regions is used for agricultural purposes and pastures where cattle and small ruminants, mainly goats, graze. 

### 4.2. Sample Preparation and Molecular Analysis

*Ixodes ricinus* ticks were collected from vegetation using the flagging method, by sweeping vegetation with flannel flags to a height of 1 m. The species and stages of the ticks were identified with the use of zoological keys [34]. 

A total of 1619 individuals of *I. ricinus* comprising all life stages—larvae, nymphs and adults—were collected. Details of the collected study material are included in Table 1. Ticks were homogenized (TissueLyser LT, QIAGEN) and DNA was isolated from each individual by the phenol-chloroform method [35]. Pools (five isolates each) were prepared for screening, following which all positive pools (PCR+ = presence of pathogen) were retested for individuals. 

Ticks were screened for the presence of DNA of *A. phagocytophilum, Rickettsia* spp. and *Francisella* spp. The following markers were used for analysis: *msp2* (gene-encoding polymorphic major outer-membrane protein) for *A. phagocytophilum*, with primers *msp3f, msp3r* [36] and *gltA* (gene-encoding citrate synthase), for *Rickettsia*, with primers RpCs877, RpCs1258 [18] and *tul4*, and for *F. tularensis*, with primers tul4-435 and tul4-863 (17-kDa lipoprotein gene primers) [37]. The expected product lengths were 334 bp (*msp2*), 382 bp (*gltA*) and 410 bp (*tul4*), respectively. For additional species characterization for *Rickettsia* spp., reactions were performed for positive samples using the *htrA* marker encoding a 17 kDa membrane protein [38]. 

All stages of ticks were tested for pathogen DNA. PCR for *A. phagocytophilum* was not performed only for *I. ricinus* larvae because it has previously been demonstrated that *Anaplasma* species do not spread through transovarial transmission [1,2]. 

PCR analysis used Color OptiTaq DNA Polymerase (EUR_X_ Ltd., Gdańsk, Poland). The PCR reaction mixture contained PCR Buffer, 2.0 mM MgCl_2_, 2.0 mM of each dNTP mix, 10 pmol/μL of forward and reverse primers and 0.2 U Taq Polymerase/10 μL master mix. The thermal-time profile was in accordance with the requirements indicated by the manufacturer. 

### 4.3. DNA Sequencing and Phylogenetic Analysis

Positive samples were sequenced for adults and larvae, and of all “PCR+” nymphs, 15 were selected for sequencing. Because no differences were found in the analyzed gene fragments, the remaining positive samples were not sequenced. The positive samples were sequenced at Macrogen Europe (Amsterdam, The Netherlands). 

Bioinformatic analyses were performed for the obtained nucleotide sequences using BLAST software and MEGA. Only the original sequences were submitted to GenBank. The Maximum Likelihood method, implemented in MEGA X, was used for phylogenetic analyses [39]. The “best fit” substitution model was calculated using the Model Test implemented in MEGA X (selected: Tamura’s 3-parameter model) [40]. 

Accession numbers: DNA sequences MZ146785 (*R. helvetica*, *gltA*) and MZ146785 (*R. helvetica*, *htrA*).

## Figures and Tables

**Table 1:** Numbers and representation of development of all stages of *Ixodes ricinus* examined in the study.

Site	Number of Ticks	Female n/%	Male n/%	Nymph n/%	Larvae n/%
**Świerznica** 21/4.42	475	14/2.9	15/3.16	252/53.05	194/40.84
**Ciemnik** 15/2.86	524	35/6.68	29/5.53	276/52.67	184/35.11
**Imno** 24/5.71	420	55/13.09	10/2.38	252/60	103/24.52
**Lubieszyn** 2/0.76	263	7/2.66	14/5.32	228/86.69	14/5.32
Total	1682	111/6.60	68/4.04	1008/59.93	495/29.43

**Table 2:** Infection of ticks by pathogens of the genera *Rickettsia* and *Anaplasma*.

Number of Ticks	Site/Infection		Female (n/%)●∆		Male (n/%)●∆		Nymph (n/%)●∆		Larvae (n/%)●∆	
	Ric n/%PCR+	Aph n/%PCR+	Ric n/%PCR+	Aph n/%PCR+	Ric n/%PCR+	Aph n/%PCR+	Ric n/%PCR+	Aph n/%PCR+	Ric n/%PCR+	Aph n/%PCR+
457	**Świerznica**		0	1/7.14	3/20.0	0	18/7.14	3/1.19	0	nt
	21/4.42	4/0.84								
524	**Ciemnik**		0	0	4/13.7	0	11/3.99	1/0.36	0	nt
	15/2.86	1/0.19								
420	**Imno**		1/1.82	0	0	2/20.0	22/8.73	2 /0.79	1/0.97	nt
	24/5.71	4/0.95								
263	**Lubieszyn**		1/14.29	0	1/7.14	0	0	0	0	nt
	2/0.76	0/0.84								
1682	Total		2/1.80	1/0.90	8/11.76	2/2.94	51/1.80	6/0.90	1/0.2	nt

Abbreviations: Ric—*Rickettsia helvetica*; Aph—*Anaplasma phagocytophiulm*; nt—not tested.

## Data Availability

https://www.ncbi.nlm.nih.gov/genbank accessed date 15 May 2021.
